# Supplementary material for: Identification of photoperiod-induced specific miRNAs in the adrenal glands of Sunite sheep (Ovis aries)
Source: Front Vet Sci. 2022 Jul 22;9:888207. doi: 10.3389/fvets.2022.888207 (PMC9354845; doi:10.3389/fvets.2022.888207)
Supplement: Supplementary file 1 [file Data_Sheet_1.ZIP › Supplementary file/miRDeep2 file.zip!/miRDeep2 file/Know_Structure/SP42A1/oar-mir-3959.pdf]

miRBase precursor : oar-mir-3959
Total read count : 16890
oar-miR-3959-5p read count : 15251
oar-miR-3959-3p read count : 1639
remaining reads : 0

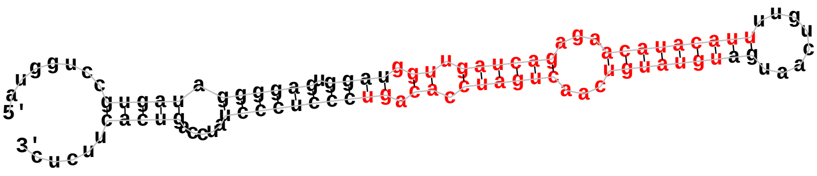

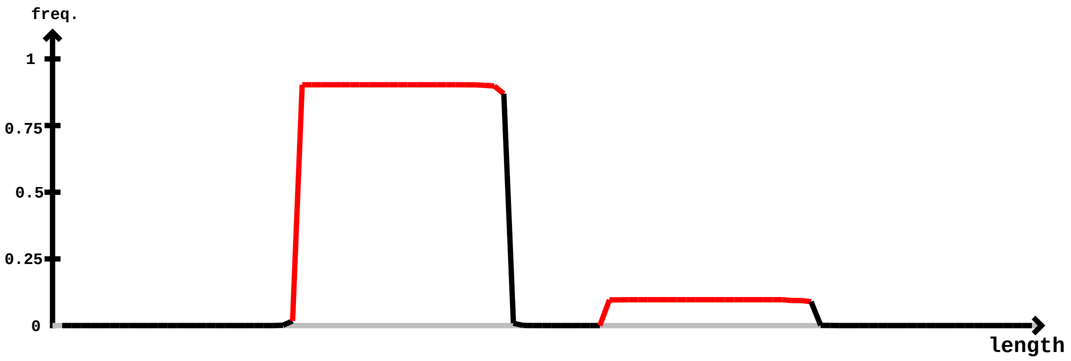


```
                                  oar-miR-3959-5p
                                                          oar-miR-3959-3p
5'- augguccgugauaggggaguggaugguugaucagagaacauacauuuugucaaugauguaugucaacugauccacagucccucccuauccagucacuucuc  -3'   exp
    .......(((((.((((((.((((.((.((((((...(((((((((........)))))))))...)))))).)).))))))))))).....))))).....   reads   mm   sample
    ......................augguugaucagagaacau............................................................   1       0    seq
    ......................augguugaucagagaacaua...........................................................   1       0    seq
    ......................augguugaucagagaacauac..........................................................   3       0    seq
    ......................augguugaucagagaacauaca.........................................................   12      0    seq
    ......................augguugaucagagaacauacauu.......................................................   2       0    seq
    .......................ugguugaucagagaacauaca.........................................................   7       0    seq
    .......................ugguugaucagagaacauacau........................................................   16      0    seq
    .......................ugguugaucagagaacauacauu.......................................................   247     0    seq
    .......................ugguugaucagagaacauacauuu......................................................   2       0    seq
    .......................ugguugaucagagaacauacauuuu.....................................................   1       0    seq
    ........................gguugaucagagaacau............................................................   1       0    seq
    ........................gguugaucagagaacaua...........................................................   2       0    seq
    ........................gguugaucagagaacauac..........................................................   29      0    seq
    ........................gguugaucagagaacauaca.........................................................   24      0    seq
    ........................gguugaucagagaacauacau........................................................   470     0    seq
    ........................gguugaucagagaacauacauu.......................................................   14285   0    seq
    ........................gguugaucagagaacauacauuu......................................................   137     0    seq
    ........................gguugaucagagaacauacauuuu.....................................................   7       0    seq
    ........................gguugaucagagaacauacauuuugu...................................................   1       0    seq
    .........................guugaucagagaacauacauu.......................................................   2       0    seq
    ..........................uugaucagagaacauacauuuu.....................................................   1       0    seq
    ........................................................uguaugucaacugauccac..........................   55      0    seq
    ........................................................uguaugucaacugauccaca.........................   2       0    seq
    ........................................................uguaugucaacugauccacag........................   70      0    seq
    ........................................................uguaugucaacugauccacagu.......................   1497    0    seq
    ........................................................uguaugucaacugauccacaguc......................   5       0    seq
    ..........................................................uaugucaacugauccacagu.......................   3       0    seq
    ..........................................................uaugucaacugauccacaguc......................   1       0    seq
    ..........................................................uaugucaacugauccacagucc.....................   6       0    seq
```
